# Molecular insights into the regulatory landscape of PKC-related kinase-2 (PRK2/PKN2) using targeted small compounds

**DOI:** 10.1016/j.jbc.2024.107550

**Published:** 2024-07-11

**Authors:** Lissy Z.F. Gross, Angelika F. Winkel, Facundo Galceran, Jörg O. Schulze, Wolfgang Fröhner, Simon Cämmerer, Stefan Zeuzem, Matthias Engel, Alejandro E. Leroux, Ricardo M. Biondi

**Affiliations:** 1 IBioBA-CONICET-MPSP, Buenos Aires, Argentina; 2 Department of Internal Medicine I, Universitätsklinikum Frankfurt, Frankfurt, Germany; 3 Department of Pharmaceutical and Medicinal Chemistry, University of Saarland, Saarbrücken, Germany

**Keywords:** chemical biology, protein kinase, PRK, PKN, PDK1, allosteric regulation, protein conformation, AGC kinases, small molecule, substrate specificity

## Abstract

The PKC-related kinases (PRKs, also termed PKNs) are important in cell migration, cancer, hepatitis C infection, and nutrient sensing. They belong to a group of protein kinases called AGC kinases that share common features like a C-terminal extension to the catalytic domain comprising a hydrophobic motif. PRKs are regulated by N-terminal domains, a pseudosubstrate sequence, Rho-binding domains, and a C2 domain involved in inhibition and dimerization, while Rho and lipids are activators. We investigated the allosteric regulation of PRK2 and its interaction with its upstream kinase PDK1 using a chemical biology approach. We confirmed the phosphoinositide-dependent protein kinase 1 (PDK1)-interacting fragment (PIF)-mediated docking interaction of PRK2 with PDK1 and showed that this interaction can be modulated allosterically. We showed that the polypeptide PIFtide and a small compound binding to the PIF-pocket of PRK2 were allosteric activators, by displacing the pseudosubstrate PKL region from the active site. In addition, a small compound binding to the PIF-pocket allosterically inhibited the catalytic activity of PRK2. Together, we confirmed the docking interaction and allostery between PRK2 and PDK1 and described an allosteric communication between the PIF-pocket and the active site of PRK2, both modulating the conformation of the ATP-binding site and the pseudosubstrate PKL-binding site. Our study highlights the allosteric modulation of the activity and the conformation of PRK2 in addition to the existence of at least two different complexes between PRK2 and its upstream kinase PDK1. Finally, the study highlights the potential for developing allosteric drugs to modulate PRK2 kinase conformations and catalytic activity.

Cells and organisms have evolved multiple regulatory mechanisms, being the phosphorylation of proteins one of the most studied mechanisms that modulate signaling pathways (1). A key component in these signaling pathways is the regulation of the enzymes that catalyze the phosphorylations, the protein kinases. PKC-related kinases (PRK1-3, also termed PKN1-3) are evolutionary related to the PKCs family and belong to the AGC group of protein kinases (2, 3). PRKs were identified as proteins interacting with small GTPases of the Rho family and are downstream effectors of Rho and Rac GTPases (4, 5, 6, 7). Downstream of Rho, PRKs mediate cell migration (8), forming part of a multiprotein signaling complex that promotes RhoA-dependent activation of p38 MAP kinase (9). PRKs are activated by arachidonic acid (10, 11). In addition, PRK2 has been found to play essential roles in hepatitis C infection (12, 13, 14, 15), different physiological situations (16, 17, 18, 19) and cancers, including prostate (20, 21, 22, 23, 24), triple-negative breast cancer (25) and cancer of the eye (26). Together, the investigations along the years have uncovered multiple roles for PRKs in healthy physiology and have highlighted PRKs as potential targets for the treatment of diverse human disorders.

Similar to PKC isoforms, PRKs possess N-terminal regulatory domains. The N-terminal region of PRKs consists of three Rho-binding domains (Hr1a, Hr1b, and Hr1c), a C2 domain, and an autoinhibitory pseudosubstrate sequence (termed PKL in PRK2). The sequence is followed by a protein kinase catalytic domain and a C-terminal region with a hydrophobic motif (HM), which in PRKs is termed the PDK1-interacting fragment (PIF) (Fig. 1, *A*–*C*). In addition, the activity of PRKs is regulated by the phosphorylation at the activation loop (Thr816 in PRK2) and Z/turn-motif sites (Thr958 in PRK2), as well as by the interaction with its upstream kinase PDK1, which phosphorylates the activation loop of PRKs (27, 28), and oligomerization, which is partly driven by the PKL pseudosubstrate-like sequence (29, 30, 31) (Fig. 1*D*).

**Figure 1:**
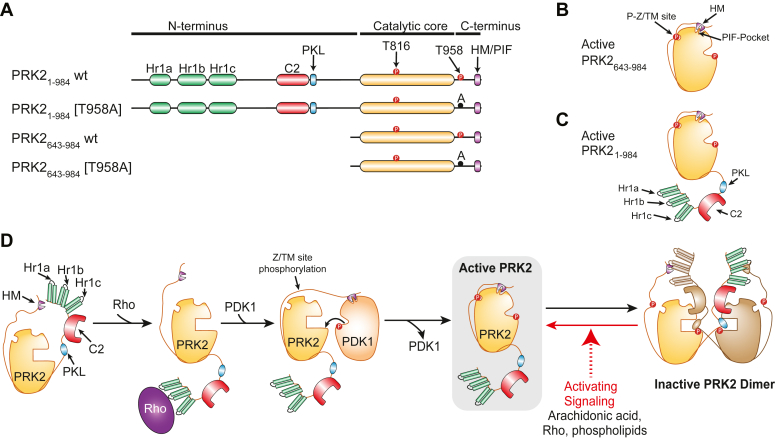
**Schematic representations of PRK2 and PRK2 N-terminal deletion proteins.***A*, the Rho-binding domains (Hr1a, Hr1b, Hr1c), the C2-like domain (C2), and the PKL sequence (PKL) are indicated as well as amino acids important for regulation and the C-terminal PIF-sequence (PIF/HM). *B*, schematic representation of the active conformation of PRK2 lacking the N-terminus (PRK2_643-984_) that is phosphorylated at the activation loop and at the phosphorylation zipper/turn-motif (P-Z/TM) site and with the hydrophobic motif (HM) docked in the PIF-pocket. *C*, the active conformation of the full-length PRK2 (PRK2_1-984_) has the same characteristics as the PRK2_643-984_ construct but with the additional N-terminal domains. *D*, the newly synthetized inactive PRK2 interacts with Rho proteins which exposes the HM/PIF sequence. The PRK2 HM (PIF) interacts with the PIF-pocket of PDK1, which allows the phosphorylation of PRK2 activation loop by PDK1. The phosphorylation of the Z/TM site triggers the interaction of the HM in *cis* and the release from PDK1, altogether achieving the active conformation. The dimerization of PRK2 leads to an inactive state, where the PLK sequence of one monomer binds on the substrate-binding site of the interacting molecule. Binding of Rho, arachidonic acid, or phospholipids releases the dimer autoinhibition. PIF, PDK1-interacting fragment; PRK, PKC-related kinase.

Aspects of the regulation of PRKs are shared with the group of AGC kinases (2, 32). Active structures of AGC kinases crystallized so far have the conserved C-terminal HM (Phe-Xaa-Xaa-Phe-Asp-Tyr) intramolecularly bound to the PIF-pocket of each kinase (Fig. 1, *B* and *C*). For most AGC kinases, the interaction between the HM and PIF-pocket is regulated and is central to the mechanism of regulation of AGC kinases (2, 33, 34). In PRKs and other AGC kinases that are substrates of PDK1 (35), the HMs are not always attached to their own PIF-pockets. In order to become phosphorylated at the activation loop (Thr816) by PDK1, the HM of PRKs first binds in *trans* to the PIF-pocket of PDK1 through a “docking-site” mediated mechanism (30, 36, 37, 38). Noteworthy, the phosphorylation at the turn motif/zipper site (T958) regulates the interaction with PDK1 (28). In addition, it is considered that the binding of the HM to the PIF-pocket of PDK1 also allosterically stabilizes the active conformation of PDK1 (Fig. 1*D*). The allostery between the PIF-pocket and the ATP-binding site of PDK1 was demonstrated also to occur in the reverse direction, as compounds and the metabolite adenosine can affect the binding of the polypeptide PIFtide (a 24 aa polypeptide comprising the sequence of PIF/the HM of PRK2) to the PIF-pocket of PDK1 (35, 39, 40). Together, the current model indicates that the PIF-pocket of PDK1 provides a docking site for the selective interaction with PRKs and other substrates and links this interaction to the activation of PDK1, thereby providing a fine-tuning mechanism for the selective and timely phosphorylation of substrates such as PRKs (4, 33, 34, 35, 36, 41, 42, 43). Lim *et al.* described that the last five amino acids of PRK2, located after the PIF/HM sequence (Phe-Arg-Asp-Phe-Asp-Tyr**-Ile-Ala-Asp-Trp-Cys-COOH**; HM underlined, five last residues of PRK2 in bold), are required for full activation by lipids and RhoA (44, 45). Interestingly, these five amino acids interact with the PDZ3 domain of the protein tyrosine phosphatase PTPN13 (46, 47).

PRK2 is also inhibited by the formation of homodimers, which are mediated by the pseudosubstrate PKL sequence interacting in *trans* with the neighbor PRK2 molecule (30) (Fig. 1*D*). However, the mechanisms by which the binding of activators release the dimer-triggered autoinhibition by the pseudosubstrate are not completely understood. The interaction of PIFtide to PRK2 allosterically displaces the binding of the inhibitory PKL sequence from the peptide substrate-binding site (30), providing evidence for the existence of an allosteric mechanism of regulation of PRK2, from the PIF-pocket regulatory site to the peptide-substrate binding site. Still, the molecular mechanisms, by which PRK2 is regulated by different stimuli or interacting proteins (CagA) (19) are still elusive.

The allosteric communication between the PIF-pocket regulatory site and the active site is not necessarily present in all AGC kinases, in particular, it is in doubt in kinases like PKA (48) or conventional PKCs (49) that are constitutively phosphorylated at the activation loop and where the regulation is given by second messengers affecting a different regulatory subunit or different regulatory domains. In these cases, the catalytic domain is considered to be in a stable-active conformation and the regulatory subunit or regulatory domains are the ones that are affected by the interaction with second messengers. In contrast to these two examples, we have extensively studied the regulation of the atypical PKC subfamily and have shown the existence of an allosteric communication between the PIF-pocket and the active site (50, 51).

In the present work, we aimed to further investigate the allosteric mechanisms governing PRK2 using a chemical biology approach. Firstly, we validate that the protein–protein interaction between PRK2 and PDK1 can be modulated with small compounds binding to the ATP-binding site of PDK1, explained by a “reverse” allosteric mechanism in PDK1. We also determined that small compounds binding to the PIF-pocket of PRK2 can allosterically activate or inhibit the intrinsic catalytic activity of PRK2. These results indicate that the allostery between the PIF-pocket and both, the ATP-binding site and the peptide-substrate binding site, plays a central regulatory role in PRKs. While the allosteric properties of PRK2 may have evolved for the physiological regulation of the kinase, the molecular insights could be exploited for future rational design of innovative drugs.

## Results

### PRK2 is activated *in vitro* by PIFtide, the HM polypeptide derived from its C terminus

**Figure 2:**
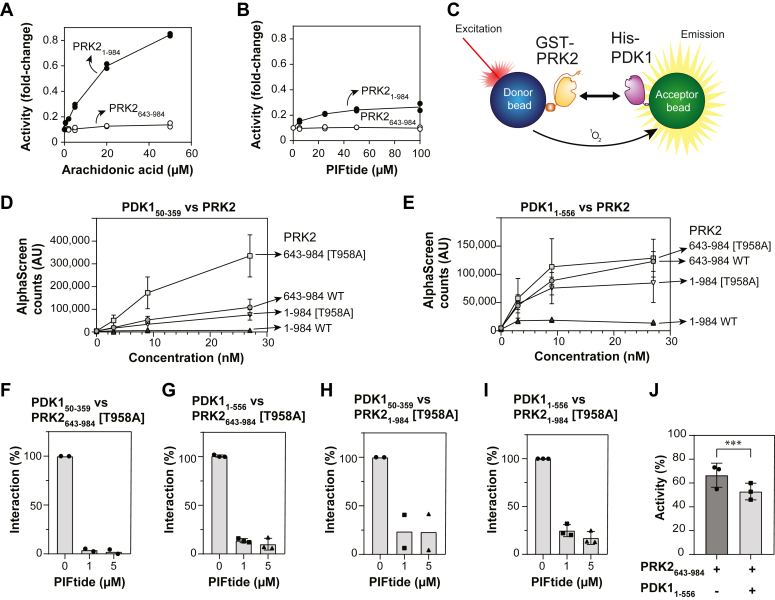
**Regulation of PRK2 activity by arachidonic acid, PIFtide, and PDK1.***A* and *B*, the kinase activity of GST-PRK2_1-984_ and GST-PRK2_643-984_ constructs was determined using KKCrosstide as the substrate. N = 2 independent experiments. *A*, the deletions constructs were activated to a lesser extent by arachidonic acid, but starting with increasing basal activities all constructs reached the activity of the isolated catalytic domain (PRK2_643-984_) in the presence of arachidonic acid. *B*, similarly to the activation by arachidonic acid, the activity of the deletion constructs are less affected by PIFtide. *C*, schematic representation of the AlphaScreen protein–protein interaction assay between GST-PRK2 and His-PDK1 constructs. *D* and *E*, comparative curves of the interaction between the PDK1 catalytic domain (PDK1_50-359_, *D*) or full-length (PDK1_1-556_, *E*) and different PRK2 constructs. N = 4 independent experiments, except the case of GST-PRK2_1-984_ WT with N = 2 independent experiments. PDK1 constructs were fixed at a concentration of 20 nM. T958A corresponds to a mutation of the Zipper/turn phosphorylation site. *F* and *I*, PDK1–PRK2 interaction specificity control by competitive displacement of PIFtide. The peptide displaced all interactions evaluated between His-PDK1, catalytic domain (PDK1_50-359_) or full-length (PDK1_1-556_), and GST-PRK2 [T958A], catalytic domain (PRK2_643-984_) or full-length (PRK2_1-984_). PDK1_1-556_ 30 nM *versus* 5 nM PRK2_643-984_ [T958A] or PRK2_1-984_ [T958A]; PDK1_1-556_ 40 nM *versus* 10 nM PRK2_643-984_ [T958A] or PRK2_1-984_ [T958A]. *F* and *H*, N = 2 independent experiments. *G* and *I*, N = 3 independent experiments. *J*, effect of the presence of 100 nM PDK1_1-556_ on the activity of 10 nM PRK2_643-984_ T958A measured using KKCrosstide as a substrate and a competitive AlphaScreen setup. N = 3 independent experiments. ∗∗∗*p* value =0.0007. A two-way ANOVA was conducted using the Geisser-Greenhouse correction to analyze the variation of the treatments and the differences between the experiments themselves. GST, glutathione-*S*-transferase; PIF, PDK1-interacting fragment; PRK, PKC-related kinase.

Our previous work showed that PIFtide, the polypeptide derived from the C-terminal region of PRK2 (Fig. 1), could allosterically inhibit the ability of the PKL region to bind to the substrate-binding site of PRK2 (30). We now tested the effect of PIFtide on the activity of PRK2. As a control, the full-length construct of PRK2 (PRK2_1-984_) was activated by arachidonic acid (Fig. 2*A*). The activation required the N-terminal domains, since the isolated catalytic domain of PRK2 (lacking the N-terminal 642 residues, PRK2_643-984_) had constitutively high activity and was not affected by arachidonic acid, as previously described (11). PRK2_643-984_ had a high specific activity that was similar to the highest specific activity achieved by the full-length construct in the presence of arachidonic acid. We now found that PIFtide activated PRK2_1-984_, although not to the full extent as arachidonic acid, while the specific activity of the catalytic domain (PRK2_643-984_) was not affected by PIFtide (Fig. 2*B*). Together with our previous findings showing the ability of PIFtide to displace the pseudosubstrate PKLtide (30), we conclude that PIFtide activates PRK2 by allosterically displacing the inhibitory pseudosubstrate PKL region. The finding also indicates that the pseudosubstrate PKL region does not bind to the active (closed) conformation of the substrate-binding site within the catalytic domain. The above underscores the significance of the allosteric coupling between the PIF-pocket and the peptide-substrate binding site in PRK2, emphasizing its role in regulation.

### Interaction of PRK2 with PDK1

By performing mutagenesis, cotransfection and pull-down experiments we previously showed that PRK2 interacts *via* the PIF-sequence with the PIF-pocket on PDK1 (28, 30, 43). The molecular details of the interaction between the HM within the PIF-sequence were also depicted in the crystal structure of the complex between PDK1_50-359_, ATP, and PIFtide (52). In order to learn more about the PRK2–PDK1 complex formation, we now established an AlphaScreen *in vitro* interaction assay between His-PDK1 and GST-PRK2 using purified wild type and mutant proteins (Fig. 2*C*). AlphaScreen allows to test the interaction of two partner molecules each binding to an acceptor or donor bead and is especially suited for testing the effect of small molecules on a given interaction. The interaction of the full-length (PDK1_1-556_) and the catalytic domain of PDK1 (PDK1_50-359_) was stronger when the PRK2 protein lacked the N-terminal region, suggesting that the N-terminal region of PRK2 decreased the interaction with PDK1. This is in agreement with the possibility that the PIF-sequence could have an alternative intramolecular binding site within the N-terminal region, as previously suggested (30). Also, the interaction with PDK1 was stronger when the turn-motif/zipper phosphorylation site of PRK2 (Thr958) was mutated to Ala (T958A) in comparison to the corresponding WT constructs (Fig. 2, *D* and *E*), as previously described using pull-down experiments (28). However, this effect was somehow lost when the constructs comprised the catalytic domain of PRK2 (643–984) and were tested against full-length PDK1 (1–556). It is interesting that we observed a distinct binding by the full-length PDK1, as we showed that the PDK1 is in equilibrium between different dimer-monomeric conformations that have distinct substrate specificities (53). The reason for the loss of effect using full-length PDK1 is not clear but could be due to the stabilization of a particular full-length conformation of PDK1, generated by the relocation of the linker-PH domain upon binding the PIF-sequence, producing a complex with decreased OFF-rate for the PIF-sequence of PRK2.

Moreover, the interactions were displaced by PIFtide (Fig. 2, *F* and *I*), in agreement with the established model where the PIF-sequence within the C-terminal region of PRK2 interacts with the PIF-binding pocket of PDK1. The high affinity interaction between PRK2 and PDK1 was obtained under conditions where PRK2 was phosphorylated at the activation loop (Fig. S1). A high affinity interaction of the phosphorylated PRK2 with PDK1 was unexpected since the simplest model suggested that, when fully phosphorylated, the C-terminal PIF/HM region of PRK2 would interact intramolecularly, as observed in all active AGC kinases crystallized so far (depicted in Fig. 1*B*).

Since we observed a high affinity interaction between the phosphorylated PRK2 and PDK1, we used this system to verify the effect of this interaction on the activity of PRK2. In this assay, we incubated the fully phosphorylated PRK2 protein with excess of PDK1. Under this condition, we ensured that the PIF-sequence of PRK2 was not intramolecularly attached to its own PIF-pocket but bound in *trans* to PDK1. Interestingly, the activity of PRK2 was only partially inhibited even in the presence of 10-fold excess of PDK1 (Figs. 2*J*; S2), indicating that the PRK2 protein held considerable catalytic activity when the PIF-sequence was not bound to its own PIF-pocket. In a previous study, we had also achieved a partial inhibition of PRK2 activity by a mAb that interacts with the C-terminal region of PRK2 (30). While both experiments showed a decrease in catalytic activity, it is noteworthy that this decrease is modest when the PIF-region is not bound to PRK2 in *cis*. This finding suggests that PRK2 may adopt the active folding conformation of the kinase domain even without the intramolecular binding of the PIF/HM sequences to their PIF-pocket.

### A PDK1 construct mutated to possess the PIF-pocket of PRK2 (PDK1 [PRK2-pocket]) has increased specific activity and cannot be further activated by PIFtide

**Figure 3:**
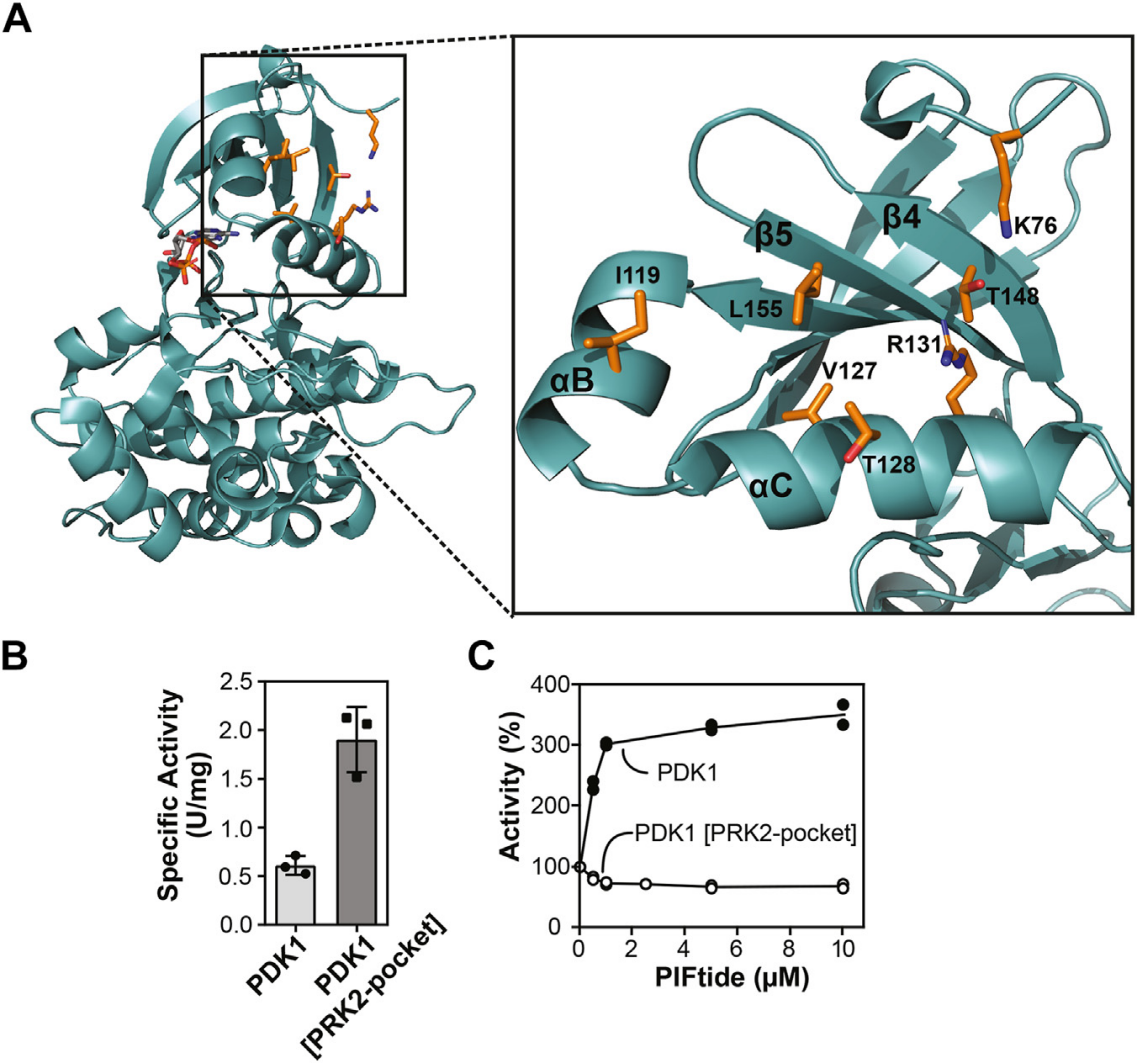
**Biochemical and structural characterization of PDK1 [PRK2-pocket].***A*, structural model of the chimeric catalytic domain of PDK1_50-359_ (*left, teal color*) and close up of the PIF-pocket (*right*). The residues mutated to resemble the PIF-pocket of PRK2 are depicted in *orange*. *B*, comparison of the specific activity of PDK1_50-359_ and the chimeric protein mimicking the PIF-pocket of PRK2 (PDK1_50-359_ [PRK2-pocket]) using T308tide as substrate. N = 3 independent experiments. *C*, effect of PIFtide on the activity of PDK1_50-359_ and the PDK1_50-359_ [PRK2-pocket] chimera. This result was obtained in at least N = 2 independent experiments with similar results. A representative result is shown with its technical duplicates as individual replicates. PIF, PDK1-interacting fragment; PRK, PKC-related kinase.

We previously crystallized the catalytic domain of PDK1 (PDK1_50-359_) and structurally investigated the effects of low-molecular-weight activators binding to its PIF-pocket by determining the crystal structures of the protein–ligand complexes, as well as by using hydrogen/deuterium exchange and fluorescence assays in solution (34). In this model, PIFtide and small compounds activators stabilize the most active conformations and drive the equilibrium toward active structures, increasing 3- to 4-fold the specific activity of PDK1 (33, 34, 54, 55, 56). In order to investigate the similarities and differences between the PIF-pockets of PDK1 and PRK2, we now mutated the PIF-pocket of PDK1_50-359_ replacing six amino acids (K76Q, I119V, V127L, T128M, R131K, T148C, and L155V) to resemble the PIF-pocket of PRK2 (Fig. 3*A*). The nonmutated construct of PDK1 had a specific activity of 0.5 U/mg (Fig. 3*B*), and this activity was increased over three-fold by PIFtide (Fig. 3*C*). Interestingly, the mutation of the residues at the PIF-pocket led to a protein with 3-fold higher specific activity (Fig. 3*B*), which was insensitive to PIFtide (Fig. 3*C*).

The finding that PDK1 [PRK2-pocket] was fully active was surprising considering the previous extensive work on PDK1 and members of other families of AGC kinases (Akt/PKB, S6K, SGK, aPKC, MSK, RSK), which in equivalent kinase assays only achieved the highest level of activity in the presence of PIFtide or HM polypeptides (Biondi *et al.* 2000; Frodin *et al.* 2002; Yang *et al.* 2002) and from the NDR/LATS family where the fusion with PIFtide rendered fully active kinases (Cook *et al.* 2014; Hoa *et al.* 2016). However, this curiosity on PRK2 was in agreement with the above finding that the *trans* interaction of the PIF-sequence from PRK2 with PDK1—rather than with its own PIF-pocket— only mildly affected the activity of PRK2. This feature was also in agreement with a previous finding where an antibody recognizing the C-terminal region of PRK2 displaced the intramolecular interaction but only modestly inhibited the activity of PRK2 (Bauer *et al.* 2012).

### Modulation of PRK2–PDK1 interaction with small compounds

**Figure 4:**
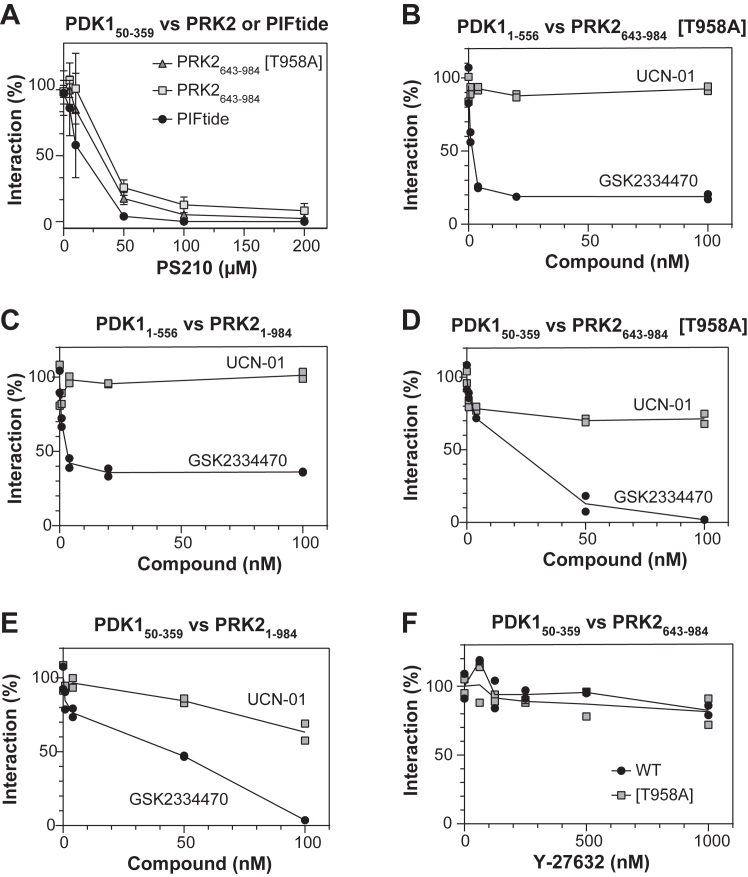
**Allosteric modulation of the PDK1–PRK2 complex.***A*, effect of the compound PS210, which binds to the PIF-pocket of PDK1, on PDK1–PRK2 complexes. A concentration-dependent displacement of the interactions is observed, equivalent to that observed in the control with the biotin-PIFtide peptide. Ten nanomolars biotin-PIFtide, 15 nM GST-PRK2_643-984_ or 15 nM GST-PRK2_643-984_ [T958A] *versus* 30 nM His-PDK1_50-359_ were used. N = 3 independent experiments. *B*–*E*, effect of PDK1 inhibitors that interact at the ATP-binding site on the interaction between PDK1 and PRK2. These results were obtained in at least N = 2 independent experiments with similar results. A representative result is shown with its technical duplicates as individual replicates. GSK2334470 inhibits by reverse allosteric modulation the interaction between the C-terminal motif of PRK2 and the PIF-pocket of PDK1, while UCN-01 has no effect (PDK1_1-556_, *B* and *C*) or has a much less potent effect (PDK1_50-359_, *D* and *E*). *B*–*C*, 30 nM PDK1 full-length (PDK1_1-556_) *versus* 5 nM PRK2_643-984_ [T958A] (*C*) or PRK2_1-984_ [T958A] (*B*). *D*, 30 nM of the catalytic domain of PDK1 (PDK1_50-359_) *versus* 20 nM PRK2_643-984_ [T958A]. *E*, 50 nM of PDK1_50-359_*versus* 50 nM PRK2_1-984_ [T958A]. *F*, the PRK2 inhibitor Y-27632 does not affect the interaction between PDK1_50-359_ and PRK2_643-984_. The interaction is performed using 30 nM of the catalytic domain of PDK1 (PDK1_50-359_) and 15 nM of the catalytic domain of PRK2 (PRK2_643-984_ both the WT and the [T958A] mutant). GST, glutathione-*S*-transferase; PIF, PDK1-interacting fragment; PRK, PKC-related kinase.

Using the above AlphaScreen interaction assay, we tested if PS210, a compound developed to bind to the PIF-pocket of PDK1, also displaced the interaction (Fig. 4*A*). Indeed, PS210 produced a displacement, reinforcing the idea that the docking between the PIF-sequence in PRK2 and the PIF-pocket of PDK1 is a key interaction site of this protein–protein complex.

Some drugs developed by the pharmaceutical industry to bind to the ATP-binding site of protein kinases produce an allosteric effect on the regulatory site (39, 57). We previously showed the distinct effect of UCN01 and GSK2334470 that bind at the ATP-binding site of PDK1 on the interaction with PIFtide (39) (Fig. S3). We therefore probed the effect of UCN01 and GSK2334470, two kinase inhibitors known to bind to the ATP-binding site of PDK1, on our interaction assay. GSK2334470 potently displaced the interaction between the different constructs of PDK1 and PRK2, while UCN01 did not affect the interaction (Fig. 4, *B*–*E*). The effect was observed both with full-length PDK1 (PDK1_1-556_) (Fig. 4, *B* and *C*) or the catalytic domain of PDK1 (PDK1_50-359_) (Fig. 4, *D* and *E*). Similarly, the disruption of the interaction was also observed when the catalytic domain of PRK2 (Fig. 4, *B* and *D*) or full-length PRK2 were tested (Fig. 4, *C* and *E*). Similar results had been observed between PDK1 and the peptide PIFtide, but it had not been shown to affect the docking interaction with the physiological substrate (39). Here, we confirmed the ability of GSK2334470 to disrupt the protein–protein complex between PDK1 and its substrate PRK2, which is explained by the “bidirectionality” of the allosteric system (39). Thus, metabolites or drugs binding to the ATP-binding site of PDK1 could affect the interaction of PDK1 with PRK2 (57). Considering these results and the allosteric nature also observed in PRK2, we wondered if the Yoshitomi compound Y-27632, an inhibitor of PRK2 that binds to its ATP-binding site, would affect allosterically the PIF-pocket and modulate the interaction with PDK1. However, in our established assay, Y-27632 did not affect the formation of the complex between the catalytic domains of PDK1 and PRK2 (Fig. 4*F*).

### Identification of small molecules directed to the PIF-pocket that can activate or allosterically inhibit PRK2

**Figure 5:**
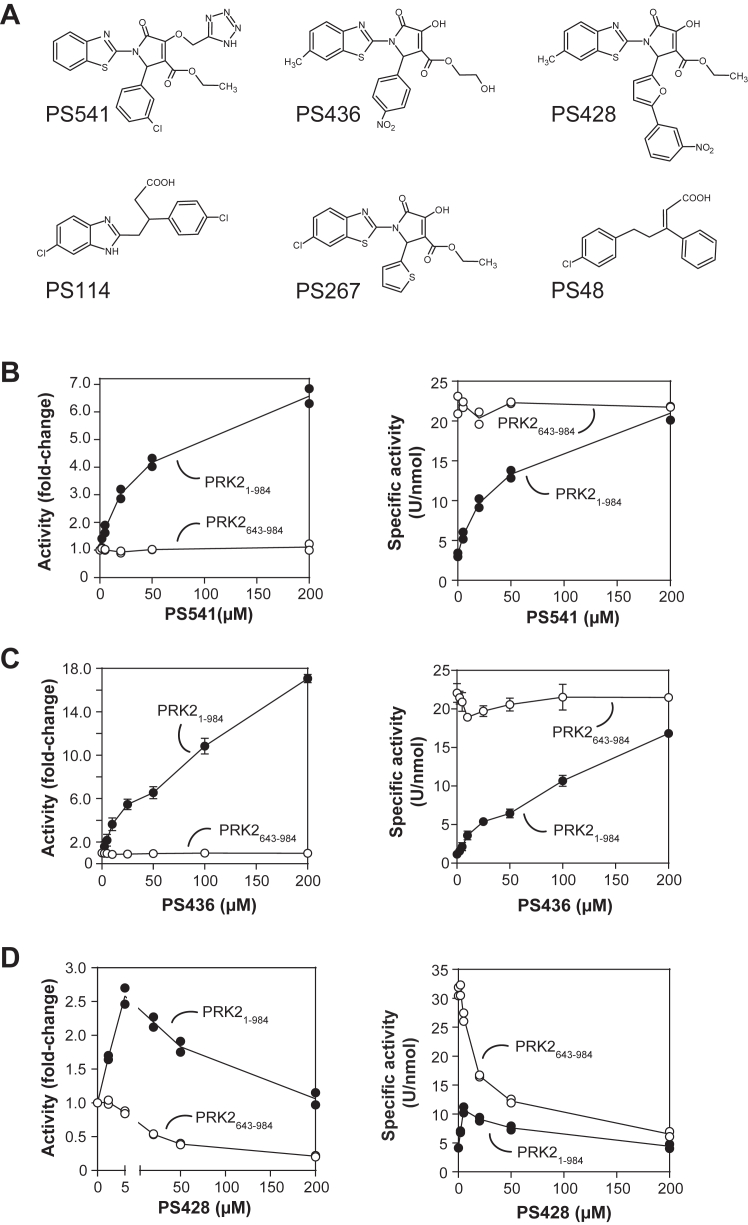
**Effect of low-molecular-weight compounds on the activity of PRK2.***A*, structures of low-molecular-weight compounds used in this study. *B*–*D*, the kinase activity of PRK2_1-984_ (*closed circles*) and PRK2_643-984_ (*open circles*) was determined using KKCrosstide as a substrate. The effect of compounds PS541 (*B*, N = 2 independent experiments), PS436 (*C*, N = 3 independent experiments) and PS428 (*D*, N = 2 independent experiments) is depicted as activity [%] (*left panel*) and specific activity [U/nmol] (*right panel*). PRK, PKC-related kinase.

Throughout the years, we developed a focused library of compounds directed to the PIF-pocket of AGC kinases comprising over 800 noncommercial small molecules (55, 58, 59, 60). The library was initially built around PDK1 activators that were identified *in silico* (55). Follow-up compounds were designed as variants of those activating PDK1, such as PS48 and PS114, (Fig. 5*A*) (34, 59, 60) and later based on scaffolds that affected the specific activity of different AGC kinases such as atypical PKCs (3, 50, 51, 58). Thus, the compounds in the focused library are either direct variants of allosteric activators or allosteric inhibitors binding to the PIF-pocket of AGC kinases, mostly PDK1 or atypical PKCs, or from different scaffolds that were designed by scaffold hopping to keep general features of active compounds with improved pharmacological properties. To investigate the role of the PIF-pocket of PRK2, we screened our focused library of compounds measuring the effect of the different compounds on the *in vitro* catalytic activity of PRK2_1-984_ and on the catalytic domain of PRK2 (PRK2_643-984_). Interestingly, we identified small compounds that activated PRK2_1-984_ and a number of other hits that inhibited PRK2 activity. Thereafter, we investigated in more depth the effect of three compounds, PS428, PS436, and PS541 on the activity of PRK2 (Fig. 5*A*).

We first evaluated in more detail the effect of the different compounds on the activity of PRK2_1-984_ and PRK2_643-984_. We confirmed that PS436 and PS541 were indeed strong activators of PRK2 (Fig. 5, *B* and *C*). Interestingly, PS541 activated PRK2_1-984_ but not the isolated catalytic domain (PRK2_643-984_), similar to the results observed with arachidonic acid (Fig. 2*A*) and PIFtide (Fig. 2*B*). The kinase activity assays performed with all PRK2 N-terminal deletion constructs revealed that addition of PS541 activated the longer PRK2 constructs up to the specific activity of the isolated catalytic domain (Fig. 5*B*). Similarly, PS436 activated PRK2_1-984_ almost up to the specific activity of the isolated catalytic domain of PRK2 (Fig. 5*C*). Although these two compounds were strong activators of the full-length PRK2, they could not further activate the isolated catalytic domain (PRK2_643-984_). This suggested that the compounds PS541 and PS436 acted like PIFtide. Moreover, the size of the small compounds indicated that the allosteric regulation was achieved by the interaction with the PIF-pocket.

Interestingly, the compound PS428 called our attention, because it activated PRK2_1-984_ at low concentrations, but inhibited this construct at higher concentrations. In contrast, the isolated catalytic domain (PRK2_643-984_) was only inhibited by this compound (Fig. 5*D*), reaching 50% of kinase inhibition at 20 μM PS428. Together, the data suggested that the PIF-pocket could mediate the allosteric activation and allosteric inhibition of PRK2.

### PS541, PS436, and PS428 activate PRK2 1-984 by binding to the PIF-pocket and allosterically displacing the N-terminal PKL sequence from the substrate-binding site

**Figure 6:**
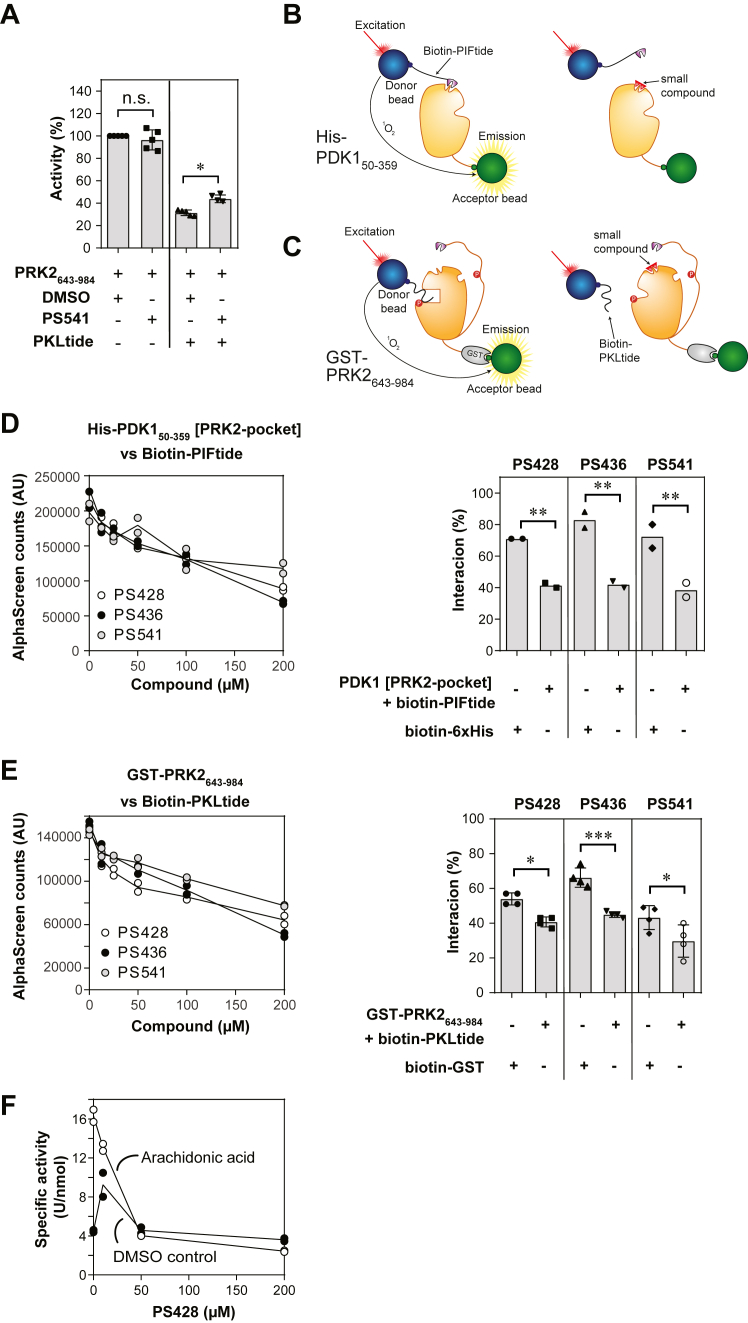
**PS541, PS436, and PS428 target the PIF-pocket of PRK2.***A*, PS541 can restore PRK2 activity inhibited by PKLtide. The activity of PRK2_643-984_ was measured with KKCrosstide as a substrate, in the presence and absence of 10 μM PKLtide and 300 μM PS541 (N = 5 independent experiments). *B*–*E* AlphaScreen experiments were carried out to investigate the binding site of the compounds. *B* and *C*, schemes of the assay setups between His-PDK1_50-359_ and biotin-PIFtide (B) or GST-PRK2_643-984_ and biotin-PKLtide (*C*). *D*, the interaction between His-PDK1 [PRK2-pocket] (30 nM) and biotin-PIFtide (30 nM) was disrupted by all tested compounds in a concentration-dependent manner (*left panel*, technical replicates, experiment performed at least twice). The signal decrease in the presence of 200 μM compound was significantly different from the biotin-6xHis control used to verify that the decrease of signal was not due to unspecific effect of the compound on the AlphaScreen technology (*right panel*); (N = 2 independent experiments). *E*, the compounds disrupted the interaction between GST-PRK2_643-984_ (1.5 nM) and biotin-PKLtide (75 nM) in a concentration-dependent manner (*left panel*, technical replicates, experiment performed at least twice) and the signal decrease in the presence of 200 μM compound was significantly different from the biotin-GST control (*right panel*). ∗*p* <0.05, ∗∗*p* < 0.01, and ∗∗∗*p* < 0.001 (N = 4 independent experiments). Significance was calculated by one-way analysis of variance followed by Bonferroni post hoc testing. *F*, in the presence of arachidonic acid (50 μM) PS428 inhibited the activity of PRK2 1-984 in a concentration-dependent manner. Technical replicates, experiment performed at least twice. GST, glutathione-*S*-transferase; PIF, PDK1-interacting fragment; PRK, PKC-related kinase.

The work by Shiga *et al.* (61) and by us (30) indicated that the mechanism of inhibition of PRKs is mediated partly by the interaction of the pseudosubstrate PKL sequence of PRK2 (PLK sequence in PRK1) with the substrate binding site of PRKs. Based on the above, we hypothesized that the small compounds, similar to PIFtide, could allosterically displace the PKL region. We tested the hypothesis in several complementing assays. In a first set of experiments, we confirmed that PS541 had no effect on the activity of PRK2_643-984_ and that it could be inhibited by 10 μM PKLtide (PKLQRQKKIFSKQQG), a polypeptide comprising the PKL region (Fig. 6*A*). Under those conditions, a high excess of PS541 reversed the inhibition of PKLtide, promoting a significant increase in activity (Fig. 6*A*). Secondly, we set up two different interaction-displacement assays using the AlphaScreen technology to study the mechanism of action of these small compounds. (Fig. 6, *B* and *C*). When a compound disrupts the interaction, there is a decrease in the light emitted by the acceptor beads. On the first AlphaScreen assay, we established the interaction between His-PDK1 [PRK2-pocket] and biotin-PIFtide (Fig. 6*B*) by varying both the concentrations of both proteins in a cross-titration assay. We selected conditions (30 nM His-PDK1 [PRK2-pocket] and 30 nM biotin-PIFtide) that provided excellent signal while still around half maximal binding. Under those conditions all three compounds displaced the interaction in a concentration-dependent manner (Fig. 6*D*). The three compounds displaced the interaction of His-PDK1 [PRK2-pocket] with biotin-PIFtide, although a small unspecific effect on the AlphaScreen system was observed on the AlphaScreen system (on the biotin-6xHis control). This result indicated that all three small molecules bound to the PIF-pocket and displaced the binding of PIFtide. In addition, the small compounds also displaced the interaction between GST-PRK2_643-984_ and biotin-PKLtide (30) (Fig. 6, *C* and *E*). We previously showed that in this assay, PIFtide allosterically displaces the binding of PKLtide from the substrate binding site. Interestingly, the three small compounds also displaced PKLtide binding to PRK2 (Fig. 6*E*).

A note of caution on the allosteric displacement of PKL by the compounds stands from our lack the conclusive knowledge of the binding site for the compounds. The compounds examined in this study were developed as part of a targeted library aimed at the PIF-pocket of AGC kinases. In addition, compounds related to the ones here investigated were previously crystallized bound to the PIF-pocket of different AGC kinases; PS541 is related to PS114 (PDB, 4A06); PS436 is related to PS267 (PDB, 5MRD) and PS428 has a third ring system like PS315 (PDB, 4CT1) (Fig. S4). Furthermore, the biochemical effects of two of the compounds, PS541 and PS436, precisely mimic the effect of PIFtide, the prototype peptide binding to the PIF-pocket. It could be argued that the displacements could be due to direct binding of the compounds to the substrate-binding site. However, our data does not fit this possibility because compounds binding to the substrate-binding site would be inhibitors of PRK2 activity, while PS541 and PS436 are activators of PRK2_1-984_ and do not affect the activity of the catalytic domain of PRK2. Together, the cumulated evidence suggest that the compounds indeed bind to the PIF-pocket, although a formal proof, such as the crystal structure of the complexes are yet to be obtained.

### PS428 allosterically inhibits activated PRK2

The above data suggested a common step in the molecular mechanism for the activation by all three compounds. However, the sole release of the N-terminal inhibition could not explain the mode of action of PS428, because this compound not only activated but in addition *inhibited* the catalytic activity of PRK2_643-984_ depending on the evaluated concentration. To further characterize the effect of PS428, we tested its effect on PRK2_1-984_ already activated by arachidonic acid (Fig. 6*F*). We observed that when PRK2_1-984_ was fully activated, PS428 specifically inhibited the activity of the kinase. Therefore, PS428 exerts an inhibitory effect only on active conformations of PRK2. Therefore, our results suggest that this compound has a dual effect: when PRK2 is a dimer/oligomer with low activity, low concentrations of PS428 would activate PRK2 by affecting the quaternary structure that inhibits the kinase. However, when PRK2 is fully active, PS428 acts solely as an allosteric inhibitor, which binds to the PIF-pocket and stabilizes an inactive conformation of the kinase in a similar manner to the compounds—PS168, PS171, PS315, and PS267—which allosterically inhibit atypical PKCs (3, 50, 51).

## Discussion

The structure of the evolutionary conserved catalytic domains of most protein kinases are known or can be modelled with high precision. However, this knowledge today falls short in providing insights into the overall protein structure, regulatory mechanisms, and dynamic interactions between the kinase and its environment. In our research, we explore the structural dynamics and regulatory mechanisms of protein kinases by investigating changes induced by their interaction with small molecules. The observed changes reflect the structural landscape, conformational flexibility and allosteric communication between sites within the protein kinase.

In our recent investigations involving PDK1, we employed this approach and demonstrated that PDK1 exists in an equilibrium among at least three conformations, including PDK1 dimers and two distinct monomeric forms. Each monomeric form is active but exhibits a different ability to phosphorylate various substrates (53). In line with this research strategy, our present study investigates the dynamics of PRK2 and its interaction with PDK1. We utilize a range of targeted molecules, such as the polypeptide PIF corresponding to the C-terminal region of PRK2, the second messenger arachidonic acid, and various small compounds binding to either the ATP-binding site or the PIF-pocket of PRK2 or PDK1. Our findings reveal a series of dynamic and orchestrated mechanisms where intermolecular and intramolecular interactions are modulated through the allosteric communication between the PIF-pocket and the active site of both interacting partners.

A notable discovery is the high-affinity interaction between phosphorylated PRK2 and PDK1, achieved even at low nanomolar concentrations. This interaction, mediated by the C-terminal region of PRK2 and the PIF-pocket of PDK1, is displaced in the presence of PIFtide. Intriguingly, this interaction does not inhibit the ability of PRK2 to phosphorylate its peptide substrate, suggesting the formation of a PRK2/PDK1 complex distinct from that involved in the phosphorylation of PRK2 at the activation loop by PDK1 and could imply an additional mechanism of regulation in a cellular context. If PRK2, when phosphorylated, interacts with PDK1 in the cell, it would prevent other substrates from being phosphorylated and activated, implying a PDK1 “sequestering” mechanism. In this case, the phosphorylation of the Zipper/turn motif would play a central role, as it would favor the interaction of the PIF-sequence intramolecularly with PRK2's own PIF-pocket, which would destabilize the PDK1–PRK2 complex and allow PDK1 to phosphorylate other substrates. A regulatory function by PRK2 could explain why the PIF-sequence has a significant higher affinity than the HM of other substrate kinases (Biondi *et al.*, 2001).

Furthermore, the PRK2/PDK1 interaction could be disrupted by small compounds binding to the PIF-pocket of PDK1 or by GSK2334470, a compound binding to the ATP-binding site of PDK1. This emphasizes the capacity of small compounds to pharmacologically modulate the formation of PDK1 complexes with physiological protein kinase substrates. Additionally, it aligns with the suggestion that metabolites could physiologically modulate protein kinase complexes by interaction with the ATP-binding site and affecting protein–protein interactions at regulatory sites (39, 57).

**Figure 7:**
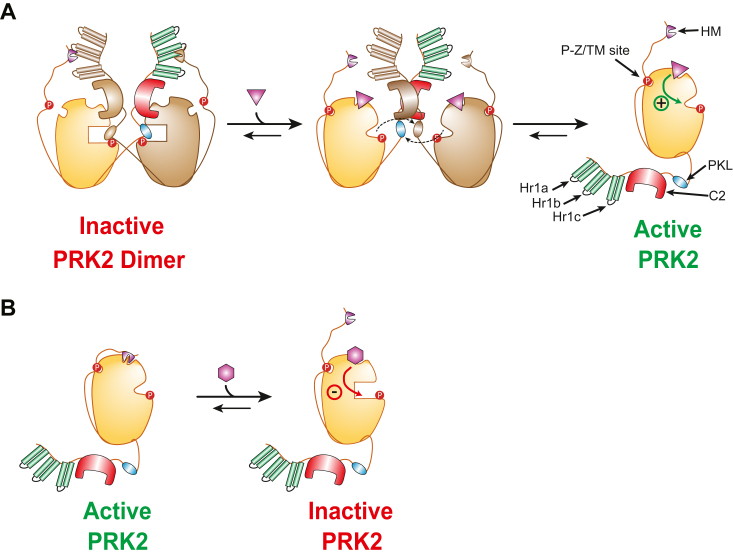
**Cartoon representation of the proposed mechanism of action for PS541, PS436, and PS428.***A*, the compounds PS541 and PS436 act as allosteric activators of PRK2 by displacing the autoinhibitory dimers and stabilizing the active monomer. *B*, proposed mechanism of action for PS428. When PRK2 is a dimer/oligomer with low activity, low concentrations of PS428 would activate PRK2 by affecting the quaternary structure that inhibits the kinase and at the same time allosterically inhibiting the kinase domain. However, when PRK2 is fully active, PS428 acts solely as an allosteric inhibitor, which binds to the PIF-pocket and stabilizes an inactive conformation of the kinase. PIF, PDK1-interacting fragment; PRK, PKC-related kinase.

Remarkably, both PIFtide and small compounds targeting the PIF-pocket of PRK2 allosterically affect the peptide-substrate binding site, releasing the inhibition caused by the PKL pseudosubstrate sequence (Fig. 7, *A* and *B*). This result supports the idea that the PIF-sequence is not bound to the PIF-pocket in the inactive conformation. Instead, intramolecular binding of PIF promotes activity by allosterically releasing the inhibition. Arachidonic acid activates PRK2 similarly to PIFtide, and this activation can be mimicked by small compounds binding to the PIF-pocket. A common mechanism for full-length PRK2 implies that physiological activators, such as lipids or interacting proteins like RhoA, induce the relocalization of the PIF-sequence to interact with the PIF-pocket, thereby releasing the inhibition exerted by the pseudosubstrate. On the same line, we can hypothesize that the stabilization of the PIF-pocket in a certain conformation could mediate the inhibition and stabilization of the pseudosubstrate-bound inactive PRK2 dimer. This model draws parallels with the role of the PIF-pocket in atypical PKCs, where the C1 domain within the N-terminal inhibitory region stabilizes an inactive conformation of the PIF-pocket (51). In this context, small compounds like PS315 were identified to bind to the PIF-pocket and allosterically inhibit PKCzeta and PKCiota catalytic activities (PDB, 4CT1) (3, 50, 51, 58). The finding that the small compound PS428, binding to the PIF-pocket, can inhibit PRK2 further supports the idea that the intrinsic dynamics of the PIF-pocket can modulate the conformation of the active site to stabilize an inactive catalytic domain, independently of the N-terminal regulatory region. An example of a very selective inhibitor stabilizing the inactive structure of the PIF-pocket is the Akt/PKB inhibitor (Akt1 1/2) (PDB, 3O96) (62) and its derivative MK-2206 that entered numerous clinical trials, as well as the selective covalent inhibitors inspired in this mechanism (63). This suggests that small compounds may be developed to bind and stabilize the pseudosubstrate-bound inactive form of full-length PRK2, although such compounds have not been described yet.

Finally, we considered interesting to suggest the interaction mode of the identified small compounds at the PIF-pocket of PRK2. The PS541 scaffold corresponds to that of PS114, a PDK1 activator we previously crystallized in complex with PDK1, (PDB, 4A06) (50) and differs in the position of the Cl atom in the benzoimidazole ring and the presence of a Cl in the benzyl group. PS541 could bind like PS114 on the PIF-pocket of PRK2, with overlapping rings in the two hydrophobic subpockets and the carboxylate interacting with the Lys131 residue (Fig. S4, *A* and *D*), similarly to the interaction between the carboxylate of PS48 with the equivalent residue, Arg131, in PDK1 (34). Thus, binding like the PS114 and PS48 activators of PDK1 explains the effect of PS541 binding to the PIF-pocket and its stabilization of the active conformation and the displacement of the PKL pseudosubstrate inhibitory sequence from the active site. PS436 scaffold on the other hand corresponds to the scaffold of PS267, which has been crystallized in complex with PDK1 [PIF-pocket PKCι] chimera (PDB, 5MRD) (3). This chemical scaffold is the result of rigidifying PS114-derivatives, keeping the angle between the two main rings systems as found in PS48 (PDB, 3HRF) (34) and PS114 crystals (50, 58) in complex with PDK1. Thus, we propose a binding of the main two ring systems similarly to the binding mode in PS267. As a major difference, PS436 does not have the negative charge; instead, PS436 has an additional arm substituted benzene rings. We predict that this extension makes interactions out from the PIF-pocket with the hydroxyl alkyl ester functionally replacing the carboxylate stabilizing the kinase regulatory spine (Fig. S4, *B* and *E*). The binding then allosterically affects the active site disfavoring the interaction with the inhibitory PKL sequence, and thereby promoting activity. On the other hand, PS428 has a third ring system. We hypothesize that without the carboxylate stabilizing the helix α-C, a deeper tunnel can be opened in between the helix α-C and the β-sheet (Fig. S4, *C* and *F*), as described for PS315 binding to the PDK1 [PIF-pocket PKCζ] chimera (51). It can be anticipated that the mechanism of allosteric inhibition may involve a similar mechanism as for PS315, that is, disrupting the Glu-Lys key salt-bridge between the Glu in helix α-C and the active site Lys, which positions the phosphates of ATP properly for catalysis.

In summary, our results underscore the catalytic domain of PRK2 as an allosteric protein, with an allosteric communication between the PIF-pocket and the active site, highlighting the potential for developing small compound allosteric activators and inhibitors of PRKs exploiting this allosteric system. Activators of PRK2 could potentially mimic its role in nutrient sensing or insulin sensitization, whereas allosteric inhibitors may find utility in the treatment of cancer or viral infections.

## Experimental procedures

### Materials

Materials for cell culture were from Greiner and Sarstedt. Human embryonic kidney 293T cells (American Type Culture collection) were cultured in 175 cm^2^ flasks in Dulbecco’s modified Eagle’s medium (Gibco) containing 10% fetal bovine serum (Sigma-Aldrich or Natocor). Media for insect cells was purchased from Invitrogen. Molecular biology techniques were performed using standard protocols. Site-directed mutagenesis was performed using a QuickChange kit (Stratagene) following the instructions provided by the manufacturer. The oligonucleotides were synthesized by Sigma-Aldrich. The DNA constructs used for transient transfection were purified from bacteria using a Qiagen plasmid Mega kit according to the manufacturer’s protocol. The plasmid DNA sequences were verified by automatic DNA sequencing (Applied Biosystems 3100 Genetic Analyzer). Protein concentration was estimated using Coomassie Plus reagent from Thermo Fisher Scientific or Bio-Rad. Antibodies were anti-GST (mouse IgG2b, G1160, Sigma), anti-PRK2 pT816 (Rabbit, sc-12891, Santa Cruz) and anti-PKC β pT641 (Rabbit, ab5785, Abcam—this antibody was used to detect pT958 of PRK2). Secondary antibodies were from LI-COR (926-68022 and 926-32213). Quantitative analysis was performed using the software Image Studio Lite (5.2, https://www.licor.com/bio/image-studio/).

### Peptides

The peptides KKCrosstide (KKGRPRTSSFAEG), PIFtide (REPRILSEEEQEMFRDFDYIADWC), and biotin-PKLtide (biotin-PKLQRQKKIFSKQQG) were synthesized by JPT Peptide Technologies. T308tide (KTFCGTPEYLAPEVRR) and biotin-PIFtide (Cys → Ser; biotin-REPRILSEEEQEMFRDFDYIADWS) were synthesized by Pepscan. phospho-KKCrosstide (PP-KKCrosstide) (KKGRPRTS(pS)FAEG) and biotin P-KKCrosstide (biotin-KKGRPRTS(pS)FAEG) were synthesized by GenScript.

### Expression and purification of protein kinases

The expression and purification of glutathione-*S*-transferase (GST)-fused proteins were performed essentially as previously described (55). GST-fused protein kinases were expressed from pEBG2T-derived plasmids by transient transfection into human embryonic kidney 293T cells utilizing a polyethylenimine method. His-PDK_150-359_ WT, His-PDK1_1-556_ WT, and His-PDK1 [PIF-pocket PRK2] were expressed in Sf9 cells using the Bac-to-Bac system from Invitrogen. Expression and purification using nickel-nitrilotriacetic acid and gel filtration was essentially performed as previously described for His-PDK1 (34). Purified recombinant GST-PRK2 constructs were fully phosphorylated at the activation look and could not be further phosphorylated by PDK1 (Fig. S1*C*), as previously determined (10, 43).

The synthesis and characterization of PS541, PS436, and PS428 are described in the Supporting information.

### AlphaScreen interaction assay

The AlphaScreen assay was performed according to the manufacturer’s protocol (PerkinElmer Life Sciences). Reactions were performed in a 25-μl final volume in white 384-well microtiter plates (Greiner). The reaction buffer contained 50 mM Tris–HCl pH 7.4 (or 20 mM Hepes pH 7.5), 100 mM NaCl, 1 to 2 mM DTT, 0.01% (v/v) Tween 20, and 0.1% (w/v) bovine serum albumin. GST-PRK2_643-984_ and biotin-PKLtide were mixed with varying concentrations of compounds or 5% dimethylsulfoxide as control. Subsequently, 5 μl of the bead solution containing anti-GST–coated acceptor beads and streptavidin-coated donor beads (20 μg/ml final concentration) was added to the reaction mixture. For the AlphaScreen with His-tagged PDK1, His-PDK1 [PIF-pocket PRK2] and biotin-PIFtide (Cys → Ser) were mixed with varying concentrations of compounds, followed by the addition of the bead solution with nickel chelate donor beads and streptavidin-coated acceptor beads (20 μg/ml final concentration). For the AlphaScreen assays between His-PDK1 and GST-PRK2 constructs the nickel chelate acceptor beads and glutathione donor beads were used (20 μg/ml final concentration). The proteins and beads were incubated in the dark for 90 min at room temperature, and the emission of light from the acceptor beads was measured in the EnVision multilabel reader (PerkinElmer Life Sciences) or the Spark reader (Tecan) and analyzed using the instrument software.

### Protein kinase activity assays

PRK2 activity was measured using a radioactive assay in a 20 μl mixture containing 50 mM Tris–HCl, pH 7.4, 0.005 mg/ml of bovine serum albumin, 0.1% (v/v) 2-mercaptoethanol, 10 mM MgCl_2_, 100 μM [-^32^P]ATP (5–50 cpm/pmol), 0.003% Brij-35, and KKCrosstide as the substrate in the presence or absence of different compounds or 5% dimethylsulfoxide as control. Similarly, PDK1 activity was measured in the same buffer using T308tide, a peptide derived from the activation loop of PKB/Akt, as the substrate. Experiments were performed in duplicates or triplicates. The results were similar in at least two separate experiments. One unit is defined as the amount of product (nmol) that is formed per minute. The catalytic activity of PRK2 was also measured using an AlphaScreen-based assay. The activity assay is performed incubating 10 nM GST-PRK2 CD WT in the presence or absence of 100 nM His-PDK1 FL at 30 °C for 30 min in a 50 μl mixture containing 50 mM Tris–HCl, pH 7.5, 0.1 mg/ml of bovine serum albumin, 0.1% (v/v) 2-mercaptoethanol, 0.01% Tween 20, 10 mM MgCl_2_, 100 μM ATP, and 100 μM KKCrosstide. The enzymatic reaction was stopped by heating the mix for 15 min at 65 °C. The amount of P-KKCrosstide generated in the enzymatic assay is determined by a competition AlphaScreen-based assay that contained: 10 nM of biotinylated P-KKCrosstide, 20 μg/ml Streptavidin donor beads, 1/500 mAb anti pSer21/9 GSK-3 α/β (Cell Signaling 9331), and 20 μg/ml protein A acceptor beads. Known amounts of P-KKCrosstide displace the interaction, reduce the detected signal, and enable to calibrate the P-KKCrosstide produced and to estimate the specific activity of PRK2 (see Fig. S2).

### Statistical analysis

All experiments were performed at least twice (N = 2 independent experiments). with similar results. For those experiments with an N = 3 or higher, the values are presented as mean ± SD. For those experiments with an N = 2, the independent values are plotted as individual replicates; in graphics presenting curves the lines connect the means. In bar graphs, each individual replicate is shown as a scatter plot. The corresponding Figure legend indicates when a representative result is shown with its technical duplicates; remaining data can be shared upon request. Significance was calculated by one-way ANOVA followed by Bonferroni post hoc testing. A *p* value <0.05 was considered statistically significant. For the activity assay performed using AlphaScreen a two-way ANOVA was conducted using N = 3 independent activity assays measured on different days to assess the effects of the days of measurement and different assay conditions. Both effects were statistically significant (days of measurement with a *p* value = 0.0007; assay conditions *p* value = 0.0081). No significant interaction was observed between the factors. GraphPad Prism (https://www.graphpad.com/) was used for the statistical analysis.

## Data availability

When representative data are presented, the remaining data can be shared upon request. All other data is contained within the manuscript.

## Supporting information

This article contains supporting information (64, 65, 66, 67, 68).

## Conflict of interest

The authors declare that they have no conflicts of interest with the contents of this article.
