# Supplementary material for: Molecular insights into the regulatory landscape of PKC-related kinase-2 (PRK2/PKN2) using targeted small compounds
Source: J Biol Chem. 2024 Jul 11;300(8):107550. doi: 10.1016/j.jbc.2024.107550 (PMC11357854; doi:10.1016/j.jbc.2024.107550)
Supplement: Supporting Information [file mmc1.pdf]

SUPPORTING INFORMATION

Molecular insights into the regulatory landscape of PKC-related Kinase-2 (PRK2/PKN2) using targeted small compounds

Lissy Z. F. Gross[1], Angelika F. Winkel[2], Facundo Galceran[1], Jörg O. Schulze[2], Wolfgang Fröhner[3], Simon Cämmerer[3], Stefan Zeuzem[2], Matthias Engel[3], Alejandro E. Leroux[1] and Ricardo M. Biondi[1,2,*]

Supporting Methods

Figure S1

Figure S2

Figure S3

Figure S4

Supporting Methods

Synthesis and characterization of PS541, PS436 and PS428

Synthesis of ethyl 4-((1*H*-tetrazol-5-yl)methoxy)-1-(benzo[*d*]thiazol-2-yl)-2-(3-chlorophenyl)-5-oxo-2,5-dihydro-1*H*-pyrrole-3-carboxylate (PS541). The synthesis of PS541 required the preparation of the building blocks (1-Trityl-1*H*-tetrazole-5-yl)methanol and PS270. (1-Trityl-1*H*-tetrazole-5-yl)methanol. The tetrazole building block (1-trityl-1*H*-tetrazole-5-yl)methanol was prepared as described in the patent WO 2007/145349 A2 with the following modification. In a 100 mL round bottom flask were placed under nitrogen 152 mg (4 mmol) $LiAlH_4$ in 5 mL anhydrous THF. A solution of 770 mg (2 mmol) ethyl 1-trityl-1*H*-tetrazole-5-carboxylate in 5 mL THF was added drop wise at 0 ºC within 10 min upon stirring. 20 min after the addition, the starting material was completely consumed (tlc). The mixture was stirred additional 40 min at room temperature the excess $LiAlH_4$ was destroyed by careful addition of 2 mL ethyl acetate. After 1 min stirring at room temperature, the mixture was poured on 100 mL 5 % citric acid. Extraction with 3 x 100 mL ethyl acetate, drying of the collected organic phases over $MgSO_4$ and concentration *in vacuo* gave the product as a fine white crystalline solid; yield: 670 mg (98 %). ^{1}H-NMR (400 MHz, $CDCl_3$) : δ = 7.35-7.29 (m, 9 H), 7.11-7.05 (m, 6 H), 4.95 (d, *J* = 6 Hz, 2 H) ppm. Ethyl 1-(benzo[*d*]thiazol-2-yl)-2-(3-chlorophenyl)-4-hydroxy-5-oxo-2,5-dihydro-1*H*-pyrrole-3-carboxylate (PS270). A mixture of 3-chlorobenzaldehyde (0.42 g, 3 mmol) and 2-amino-benzothiazole (0.5 g, 2.7 mmol) was dissolved in 10 mL toluene, and about 10 drops of acetic acid as a catalyst were added. The mixture was stirred overnight under reflux at 105 °C, yielding a clear yellow solution. The solvent was removed by rotary evaporation, and the residue washed with diethyl ether and dried to yield the imine intermediate as a cream-colored solid; yield: 0.44 g (60%). This intermediate (414 mg, 1.5 mmol) was dissolved together with diethyl oxaloacetate sodium salt (346 mg, 1.55 mmol) in 14 mL ethanol, and the solution stirred for 1 h under reflux. Decolorization of the solution was noted, followed by the formation of a precipitate. 14 mL of a 1M HCl solution were added, and the stirring was continued for 30 min at room temperature. The crystalline precipitate was sucked off, washed twice with 4 mL 1M

HCl, then with 6 mL ethanol and dried. Thus the title compound PS270 was obtained as a white solid; yield: 432 mg (67 %), purity (HPLC): 95.2%. ^{1}H-NMR (500 MHz, DMSO-d6): δ = 1.109 (t, *J* = 7 Hz, 3 H), 3.99-4.133 (m, 2 H), 6.10 (s, 1 H), 7.28-7.36 (m, 3 H), 7.37-7.41 (m, 1 H), 7.51-7.53 (m, 1 H), 7.64 (d, *J* = 7 Hz, 1 H), 7.99 (d, *J* = 7 Hz, 1 H); ^{13}C-NMR and DEPT (500 MHz, DMSO): δ = 13.84 (CH_3), 60.05(CH_2), 60.88(CH), 113.93(C), 121.17(CH), 121.92(CH), 124.22 (CH), 126.26(CH), 126.37(CH), 127.99(CH), 128.05(CH), 129.95(CH), 131.27(C), 132.50(C), 139.16(C), 148.08(C), 151.58(C), 154.50(C), 161.54 (CO), 164.46(CO); MS (+ESI) (m/z): [M+] calcd. 414.87; found, 414.84.

Mitsunobu reaction of the pyrrolidindione PS270 with 1-triphenylmethyl-1H-tetrazole-5-methanol. In a 25 mL round bottom flask were dissolved under nitrogen 415 mg (1 mmol) pyrrolidindione PS270, 319 mg (1.2 mmol) triphenylphosphine and 342.4 mg (1 mmol) 1-triphenylmethyl-1*H*-tetrazole-5-methanol in 27 mL THF. The solution was cooled to 0 ºC and 242.7 mg (1.2 mmol) DIAD was added. The mixture was stirred 20 h at room temperature, then the solvent was removed *in vacuo* and the residue was purified by chromatography on SiO_2 with hexane: ethyl acetate = 5 : 1 (v/v). The product, ethyl 1-(benzo[*d*]thiazol-2-yl)-2-(3-chlorophenyl)-5-oxo-4-((1-trityl-1*H*-tetrazol-5-yl)methoxy)-2,5-dihydro-1*H*-pyrrole-3-carboxylate, was obtained as a white solid; yield: 500 mg (68 %). ^{1}H-NMR (400 MHz, $CDCl_3$) : δ = 1.23 (t, *J* = 7 Hz, 3 H), 4.01 (s, 2 H), 4.17 (q, *J* = 7 Hz, 2 H), 7.07-7.13 (m, 8 H), 7.28-7.37 (m, 15 H). *Cleavage of the trityl group.* In a 25 mL round bottom flask were dissolved 100 mg ethyl 1-(benzo[*d*]thiazol-2-yl)-2-(3-chlorophenyl)-5-oxo-4-((1-trityl-1*H*-tetrazol-5-yl)methoxy)-2,5-dihydro-1*H*-pyrrole-3-carboxylate in 10 mL anhydrous methanol. 31.2 mg (0.27 mmol) pyridine hydrochloride was added and the mixture was heated to 50 ºC for 2 h. Then it was poured on a small volume (10 mL) of water, acidified with HCl to pH 2-3 and extracted with 4 x 25 mL of ethyl acetate. The collected organic phases were dried over $MgSO_4$ and concentrated *in vacuo*. The residue was purified by recrystallization from methanol to give the final compound PS541; yield: 54 mg (80 %); purity (HPLC): 99.0%. ^{1}H-NMR (500 MHz, DMSO-d6) : δ = 1.036 (t, *J* = 7 Hz, 3 H), 3.97-4.09 (m, 2 H), 6.06-6.12 (m, 2 H), 6.192 (s, 1 H), 7.31-

7.38 (m, 2 H), 7.39-7.43 (m, 1 H), 7.46-7.49 (m, 1 H), 7.62- 7.64 (m, 1 H), 7.64-7.66 (m, 1 H), 8.01-8.02 (m, 1 H); ^{13}C-NMR (500 MHz, DMSO): δ = 13.5(CH_3), 32.3 (CH), 60.85(CH_2), 61.15(CH), 62.46(CH_2), 121.25(CH), 121.99(CH), 122.15(C), 124.28(CH), 126.47(CH), 127.00(CH), 128.02(CH), 128.36(CH), 129.96(C), 131.24(C), 132.71(C), 137.96(C), 147.97(C), 149.84(C), 154.25(C), 160.36(CO), 163.27(CO); MS (+ESI) (m/z): [M+] calcd. 496.93; found, 496.88.

Synthesis of ethyl 4-hydroxy-1-(6-methylbenzo[*d*]thiazol-2-yl)-2-(5-(3-nitrophenyl)furan-2-yl)-5-oxo-2,5-dihydro-1*H*-pyrrole-3-carboxylate (PS428). A mixture of 5-(3-nitrophenyl)furfural (109 mg, 0.5 mmol) and 2-amino-6-methylbenzothiazole (82 mg, 0.5 mmol) was dissolved in 2 mL toluene, and two drops of acetic acid as a catalyst were added. The mixture was stirred for 45 min under reflux at 105 °C, then the solvent was removed by evaporation. To the crude imine intermediate, 4 mL of a 0.125 M solution of diethyl oxaloacetate sodium salt in ethanol were added, and the solution stirred for 1 h under refux. Then 1 mL of 1 M HCl and 5 mL water were subsequently added to precipitate the product. The crystalline precipitate was sucked off, washed twice with 0.5 mL 1M HCl, then with 1 mL ethanol and 1 mL of diethyl ether and left in the air to dry. Thus, the title compound PS428 was obtained as a yellow powder; yield: 101 mg (40%); purity (HPLC): 96.4 %. ^{1}H-NMR (500 MHz, DMSO-d6): δ = 1.25 (t, *J* = 7 Hz, 3 H), 4.17-4.39 (m, 2 H), 6.45 (s, 1 H), 6.91 (d, *J* = 4 Hz, 1 H), 7.28 (d, *J* = 3 Hz, 1 H), 7.35 (dd, *J* = 2 Hz, *J* = 8 Hz, 1 H), 7.76 (d, *J* = 8 Hz, 1 H), 7.80 (t, *J* = 8 Hz, 1 H), 7.91-7.92 (m, 1 H), 8.13 - 8.15 (m, 1 H), 8.2-8.22 (m, 1 H), 8.45-8.46 (m, 1 H); ^{13}C-NMR (500 MHz, DMSO): δ = 13.95(CH_3), 20.94(CH_3), 55.27(CH), 60.08(CH_2), 109.48(CH), 111.22(C), 112.29(CH), 117.29(CH), 120.88(CH), 121.47(CH), 121.83(CH), 127.75(CH), 129.29(CH), 130.61(CH), 131.39(C), 131.44(C), 133.88(C), 146.17(C), 148.37(C), 149.81(C), 149.84(C), 151.71(C), 153.74(C), 161.58(CO), 164.00(CO); MS (+ESI) (m/z): [M+] calcd. 505.51; found, 505.88.

Synthesis of 2-hydroxyethyl 4-hydroxy-1-(6-methylbenzo[*d*]thiazol-2-yl)-2-(4-nitrophenyl)-5-oxo-2,5-dihydro-1*H*-pyrrole-3-carboxylate (PS436). Ethyl 4-hydroxy-1-(6-methylbenzo[*d*]thiazol-2-yl)-2-(4-nitrophenyl)-5-oxo-2,5-dihydro-1*H*-pyrrole-3-carboxylate was

first synthesized as described for PS428, using 0.82 g (5 mmol) 2-amino-6-methylbenzothiazol, 0.76 g (5 mmol) 4-nitrobenzaldehyde, dissolved in 20 mL toluene with 20 drops of acetic acid as a catalyst, and 1.05 g (5 mmol) diethyl oxaloacetate sodium salt (added again as a 0.125 M solution in ethanol). The finally obtained precipitate (slightly yellow flakes, 1.81 g, 83% yield) was used without further purification for the next step: 0.44 g (1 mmol) was dissolved in 12 mL of a dioxane/ethylenglycol mixture (2:1, v/v) while heating. 0.25 mL (1 mmol) of 4 M HCl in dioxane was added, and the mixture was stirred overnight at 100 °C. Then the mixture was cooled to room temperature, poured on 50 mL water, and the precipitate sucked off. After washing with water, ethanol and diethyl ether, the residue was dried *in vacuo*. Thus, PS436 was obtained as a light yellow solid, yield: 0.35 g (77%); purity (HPLC): 95.4 %. ^{1}H-NMR (500 MHz, DMSO-d6): δ = 2.51 (s, 3 H), 3.64-3.68 (m, 2 H), 4.08-4.14 (m, 2 H), 4.18-4.22 (m, 2 H), 6.39 (s, 1 H), 7.32 (dd, *J* = 2 Hz, *J* = 9 Hz, 1 H), 7.62 (d, *J* = 8 Hz, 1 H), 7.86 (d, *J* = 8 Hz, 2 H), 7.91 (s, 1 H), 8.27 (d, *J* = 8 Hz, 2 H); ^{13}C-NMR (500 MHz, DMSO): δ = 20.91(CH_3), 58.71(CH_2), 0.54(CH), 65.85(CH_2), 113.52(C), 120.81(CH), 121.45(CH), 123.22(CH), 127.71(CH), 129.22(CH), 131.38(C), 133.91(C), 144.59(C), 145.96(C), 147.08(C), 151.98(C), 153.65(C), 161.48(CO), 164.24(CO); MS (+ESI) (m/z): [M+] calcd. 455.44; found, 455.81.

Figure S1

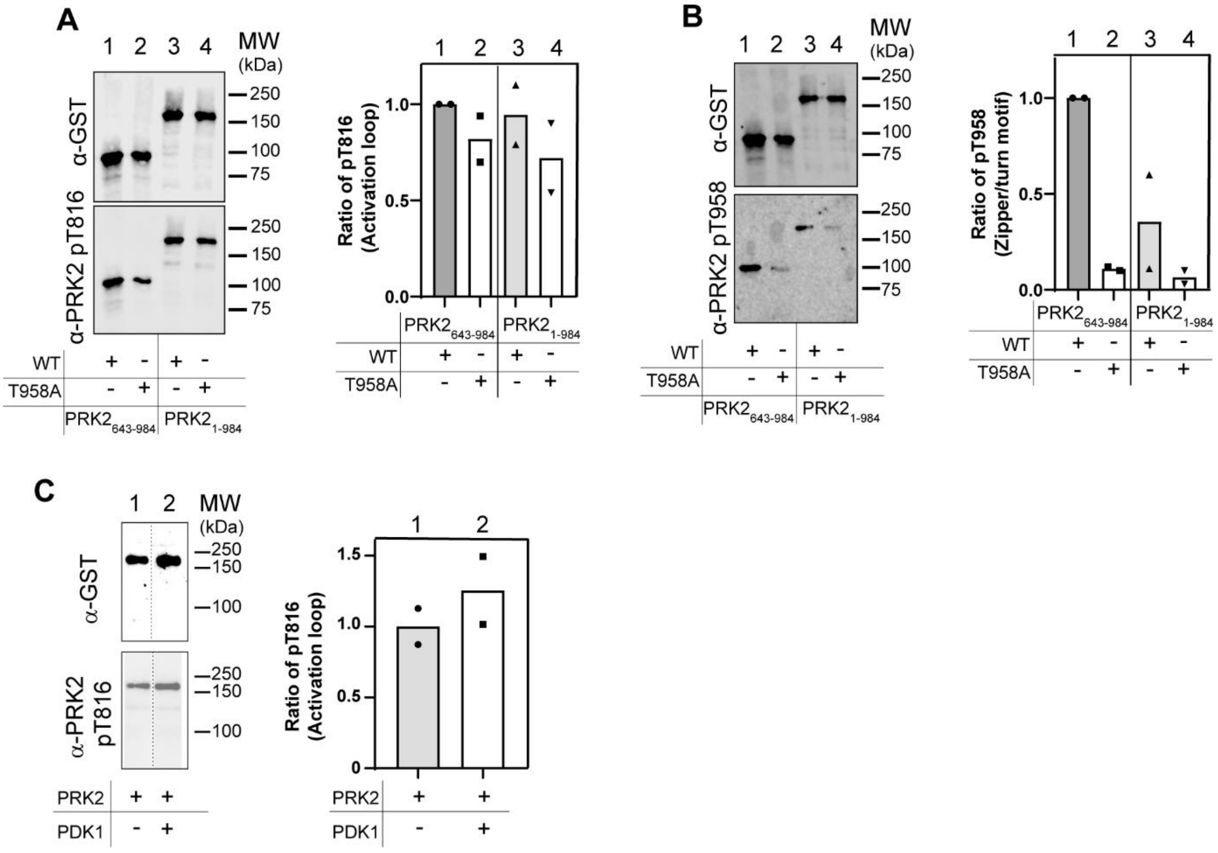

Figure S1. Phosphorylation state of PRK2 employed in the studies. (*A*-*B*) Western Blots of the phosphorylation state of the used PRK2 constructs. An anti- GST antibody is used as a loading control. The membranes are shown on the left and the quantification of the fluorescence signal on the right, each relativized to its loading control and taking the signal of the native PRK2 catalytic domain protein ($PRK2_{643-984}$ WT) as a relative value of 1.0 for reference. The blots were performed twice with different lots of purified proteins (N=2). *A*, Western blot with phosphospecific Thr816 antibody (pT816), which corresponds to the activation loop. It can be observed that all four constructs have the activation loop phosphorylated, although the catalytic domain mutated at the Zipper/turn site (T958A) has a slightly lower phosphorylation signal than the rest. *B*, Western blot with a phosphospecific Thr958 antibody (pT958), which corresponds to the Zipper/turn motif. The full-length native PRK2 protein has a 60% of the phosphorylation signal compared to the catalytic domain. C, $PRK2_{643-984}$ WT was incubated in the presence or absence of $PDK1_{1-556}$ with 10 mM $MgCl_2$ and

0.1 mM ATP at 30 ºC for 30 min. The level of PRK2 activation loop phosphorylation was assesed by Western Blot using a phosphospecific antibody and an anti-GST antibody as a loading control. The experiments were performed twice with similar results. A representative blot is shown that is a composite of two lanes which were originally separated by a molecular weight marker. To simplify the figure interpretation, the molecular weight marker lane was removed and the truncated region marked by a dotted line. The bar graphs present the quantification of the phosphorylation signals of the two experiments normalized to its loading control.

Figure S2

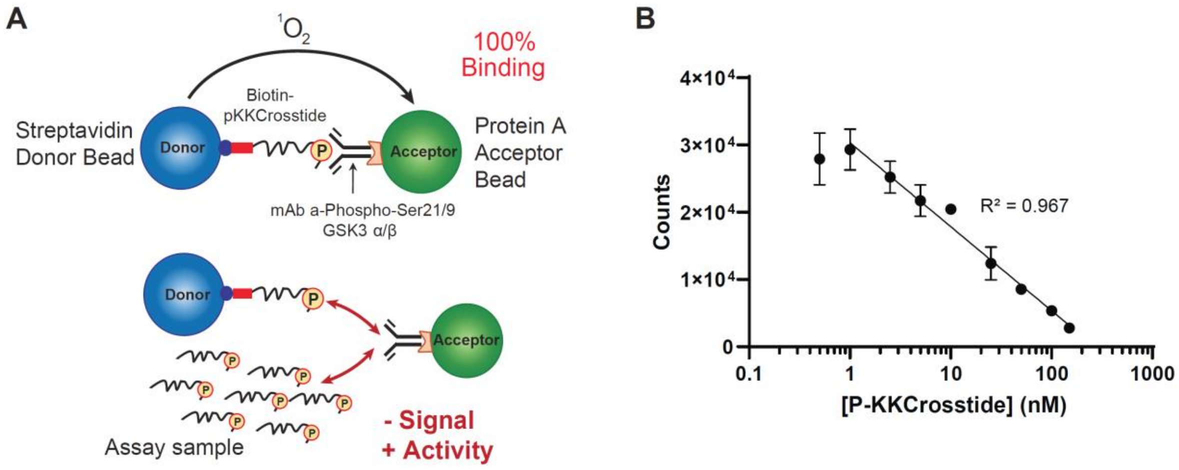

Figure S2. Detection of the phosphorylation of KK-crosstide by PRK2 using an Alphascreen competition assay. A, The alphascreen assay contains 10 nM biotinylated p-KKCrosstide, 20 µg/ml Streptavidine donor beads, 1/500 mAb anti pSer21/9 GSK-3 α/β, and 20 µg/ml Protein A acceptor beads. In the absence of any exogenous p-KKCrosstide, the assay gives the maximum signal corresponding to the 100% peptide binding. The addition of the p-KKCrosstide, generated in the previous enzymatic assay, to the alphascreen mix displaces the binding of the biotinylated p-KKCrosstide to the Protein A acceptor beads reducing the alphascreen signal. B, The calibration of the competition assay using known

amounts of p-KKCrosstide showed that the system is suitable for detecting phosphorylated peptide concentrations between 1 and 150 nM.

Figure S3

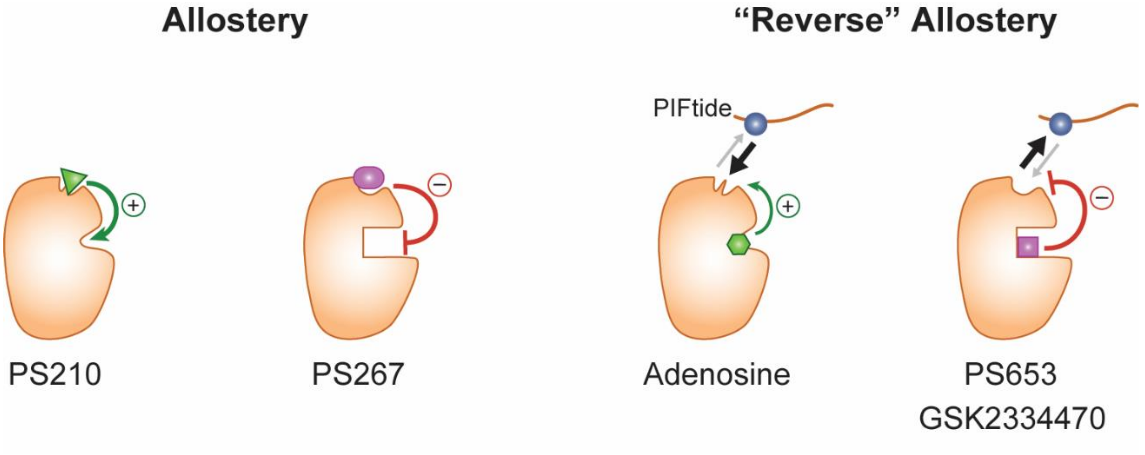

Figure S3. Bi-directional allostery in AGC kinases. Schematic representation of the binding site of small compounds PS210, PS267, adenosine and PS653/GSK2334470 and their allosteric effects, indicated by arrows. Both PS210 (PDB: 4AW1) and PS267 (PDB: 5MRD) bind to the PIF-pocket of AGC kinases PDK1 and atypical PKCs (Arencibia et al. 2017; Busschots et al. 2012). PS210 binds to the PIF-pocket of PDK1 and, like PIFtide, allosterically stimulates catalytic activity, whereas the binding of PS267 to the PIF-pocket of the atypical PKCs allosterically inhibits kinase activity. These compounds displace the binding to PIFtide, by competition with the PIF-pocket binding site. Other compounds that target the PIF-pocket and allosterically regulate the kinase activity, such as PS48 and RS1, have also been described (Bobkova et al. 2010; Engel et al. 2006; Frohner et al. 2011; Hindie et al. 2009; Kroon et al. 2015; Lopez-Garcia et al. 2011; Pastor-Flores et al. 2013; Rettenmaier et al. 2014; Sadowsky et al. 2011; Stroba et al. 2009; Xu et al. 2019; Zhang et al. 2014); The allosteric inhibitor VIII of Akt/PKB (Akti1-2; PDB: 3O96) (Wu et al. 2010), and its follow-up drugs in clinical trials (i.e. MK2206) also bind at the PIF-pocket regulatory site in the inactive conformation of the molecular switch and allosterically affect the active site (as represented for PS267) (Leroux et al. 2018). Adenosine (PDB: 5LVN), PS653 (PDB: 5LV), and GSK2334470 bind at the ATP-binding site of PDK1, but they differ in their allosteric effects on the PIF-pocket. Adenosine stimulates protein-protein interactions at the PIF-pocket, whereas PS653 and the potent PDK1

inhibitor GSK2334470 disrupts the interaction with PIFtide, in a process that we have termed “reverse” allostery (Ghode et al. 2020; Leroux and Biondi 2020; Schulze et al. 2016). In contrast to GSK2334470, another potent ATP-competitive inhibitor of PDK1 termed UCN-01 does not affect protein-protein interactions at the PIF-pocket (Schulze et al. 2016).

Figure S4

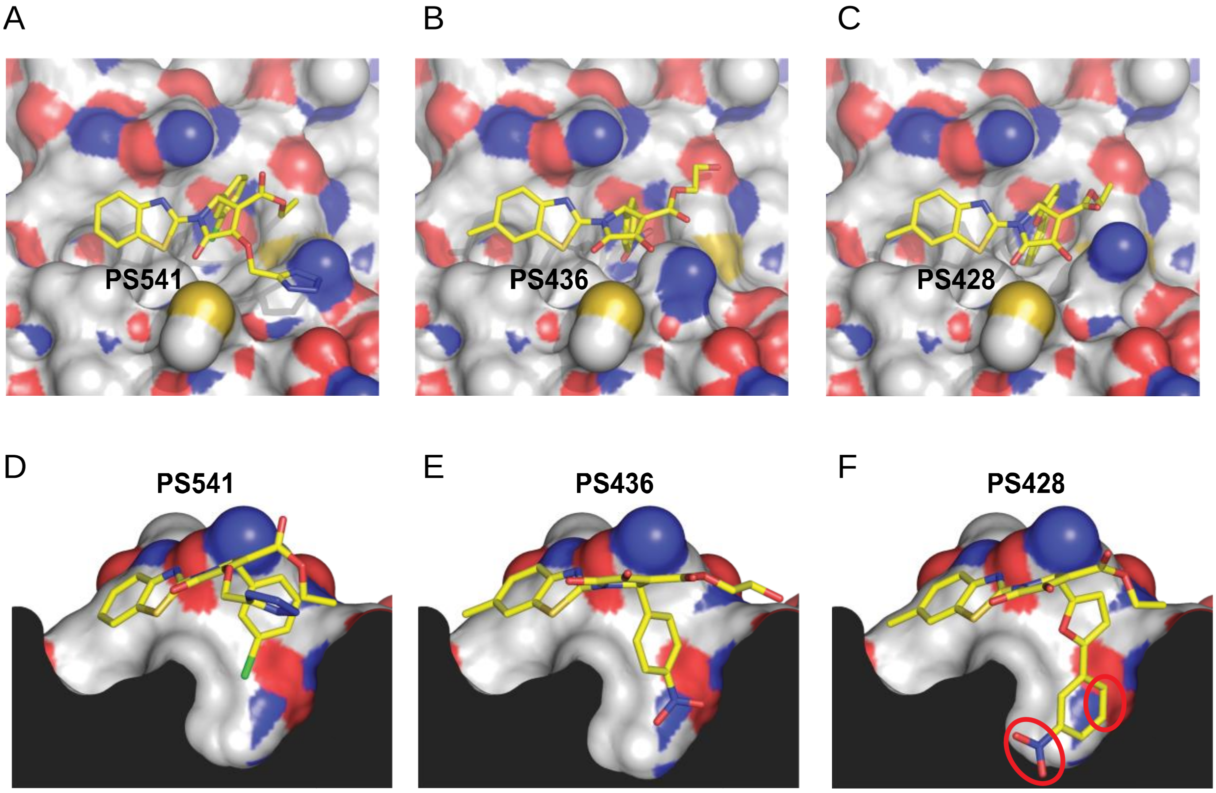

Figure S4. Suggested binding mode of the allosteric compounds in the PIF-pocket of PRK2. *A and D*, PS541; B and E, PS436; C and F, PS428. The binding mode of PS541 was modeled based on PS114, a PDK1 activator corresponding to the same scaffold that we previously crystallized in complex with PDK1, (PDB, 4A06) (Lopez-Garcia et al. 2011). The binding mode of PS436 was modeled based on the crystal structure of PS267 in complex with PDK1[PIF-pocket PKCι] chimera (PDB, 5MRD) (Arencibia et al. 2017). PS428 has a third ring system, like PS315 (PDB, 4CT1) (Zhang et al. 2014). We suggest that PS428 could bind deeper into the pocket, like PS315, which was crystallized in complex with PDK1[PIF-pocket PKCζ] chimera (Zhang et al. 2014).
